# Controlling Adult Stem Cell Behavior Using Nanodiamond-Reinforced Hydrogel: Implication in Bone Regeneration Therapy

**DOI:** 10.1038/s41598-017-06028-y

**Published:** 2017-07-26

**Authors:** Settimio Pacelli, Ryan Maloney, Aparna R. Chakravarti, Jonathan Whitlow, Sayantani Basu, Saman Modaresi, Stevin Gehrke, Arghya Paul

**Affiliations:** 1 BioIntel Research Laboratory, Department of Chemical and Petroleum Engineering, Bioengineering graduate program, School of Engineering, University of Kansas, Lawrence, KS 66045 USA; 2 Department of Chemical and Petroleum Engineering, University of Kansas, Lawrence, KS 66045 USA

## Abstract

Nanodiamonds (NDs) have attracted considerable attention as drug delivery nanocarriers due to their low cytotoxicity and facile surface functionalization. Given these features, NDs have been recently investigated for the fabrication of nanocomposite hydrogels for tissue engineering. Here we report the synthesis of a hydrogel using photocrosslinkable gelatin methacrylamide (GelMA) and NDs as a three-dimensional scaffold for drug delivery and stem cell-guided bone regeneration. We investigated the effect of different concentration of NDs on the physical and mechanical properties of the GelMA hydrogel network. The inclusion of NDs increased the network stiffness, which in turn augmented the traction forces generated by human adipose stem cells (hASCs). We also tested the ability of NDs to adsorb and modulate the release of a model drug dexamethasone (Dex) to promote the osteogenic differentiation of hASCs. The ND-Dex complexes modulated gene expression, cell area, and focal adhesion number in hASCs. Moreover, the integration of the ND-Dex complex within GelMA hydrogels allowed a higher retention of Dex over time, resulting in significantly increased alkaline phosphatase activity and calcium deposition of encapsulated hASCs. These results suggest that conventional GelMA hydrogels can be coupled with conjugated NDs to develop a novel platform for bone tissue engineering.

## Introduction

Nanocomposite hydrogels have recently become an exciting subject of study for tissue engineering applications^1–3^. These scaffolds represent a combination of polymers and other nanomaterials that display unique features compared to conventional hydrogels^4–6^. Specifically, the presence of nanomaterials can modulate the physical and mechanical properties of scaffolds^7–9^ and guide the processes of cellular adhesion and proliferation^10^. In addition, they can be used as carriers for loading therapeutics through covalent linking^11^, electrostatic interactions^12^, or physisorption^13, 14^. All of these concepts are imperative particularly for the design of bioactive bone scaffolds^15^. In fact, nanomaterials can not only function as crosslinkers within the hydrogel network to provide additional mechanical support, yet they can also modulate the release of bioactive molecules that enhance the process of bone healing^16, 17^. Based on these concepts, a variety of nanofiller agents have been proposed, ranging from silicate nanoparticles^12, 18^ to carbon-based materials^19^, to improve the mechanical properties of conventional hydrogels. Among these, an emerging option are nanodiamonds (NDs) which offer a series of advantages for modulating physical and biological properties of available hydrogels for bone regeneration^20, 21^. One of the main characteristics of NDs is the large variety of functional groups that can be introduced on their surface. Consequently, several synthesis techniques have led to the creation of carboxylated NDs, amine-functionalized NDs, or fluorescent NDs, among many others^22, 23^. The tailorable surface of NDs has allowed researchers to demonstrate delivery of different therapeutics, including small molecules^24, 25^, proteins^26–29^, and nucleic acids^30, 31^. In addition to their flexible drug delivery capabilities^32, 33^, several *in vitro* studies have shown that NDs are significantly less toxic to many cell lines than other carbon nanomaterials such as graphene and single- and multi-walled carbon nanotubes^34–37^. Moreover, a significant number of *in vivo* biodistribution studies demonstrated that NDs could be excreted over time with negligible tissue damage^38–41^. Along with the advantages mentioned above, NDs have also been investigated as a filling agent to improve the mechanical properties of biodegradable poly(L-lactic acid) scaffolds showing the possibility to increase the compressive strength of these systems to near that of cancellous bone^20, 42–44^.

Based on these encouraging studies, it is evident that NDs represent a possible strategy to modulate physical and biological properties of current scaffolds and further extend their application as bone regenerative materials. An interesting example is represented by gelatin methacrylamide (GelMA) hydrogels, which have shown excellent biocompatibility in many previous studies^45, 46^. Furthermore, GelMA has been used as a versatile platform for emerging biomedical technologies, such as in microscale photo-patterning and bioprinting. Despite these promising features, GelMA hydrogels lack the necessary mechanical and osteogenic properties that are required for the design of successful scaffolds for bone regeneration.

Our hypothesis is that NDs can be used both as a nanofiller agent and as a carrier for dexamethasone (Dex) to influence the mechanical properties and osteogenic potential of GelMA hydrogels. To test our hypothesis, we prepared and characterized the complex between NDs and Dex and tested its bioactivity with human adipose stem cells (hASCs) by studying their gene and focal adhesion expression. Prior to the design of the nanocomposite hydrogel, we assessed the *in vitro* biocompatibility of NDs on hASCs as well as using a zebrafish embryogenesis nanotoxicity assay as an *in vivo* model. We further evaluated the impact of NDs on the physical and mechanical properties of GelMA networks by monitoring the change in substrate stiffness as a result of the inclusion of NDs. We also investigated whether this change in substrate stiffness could influence traction forces of hASCs in 2D culture. Subsequently, we tested the drug delivery capabilities of NDs by monitoring the release of Dex from the nanocomposite hydrogels to determine whether the presence of NDs in the GelMA scaffold could extend drug retention. Finally, we assessed whether or not the inclusion of ND-Dex complexes within the GelMA scaffold was capable of enhancing osteogenic differentiation of encapsulated hASCs with respect to GelMA scaffolds loaded with only Dex. Overall this study sheds light on the efficacy of NDs in modulating the mechanical properties of GelMA hydrogels and their ability to control osteogenic differentiation of hASCs.

## Results and Discussion

### Preparation and characterization of the ND-Dex complex

**Figure 1:**
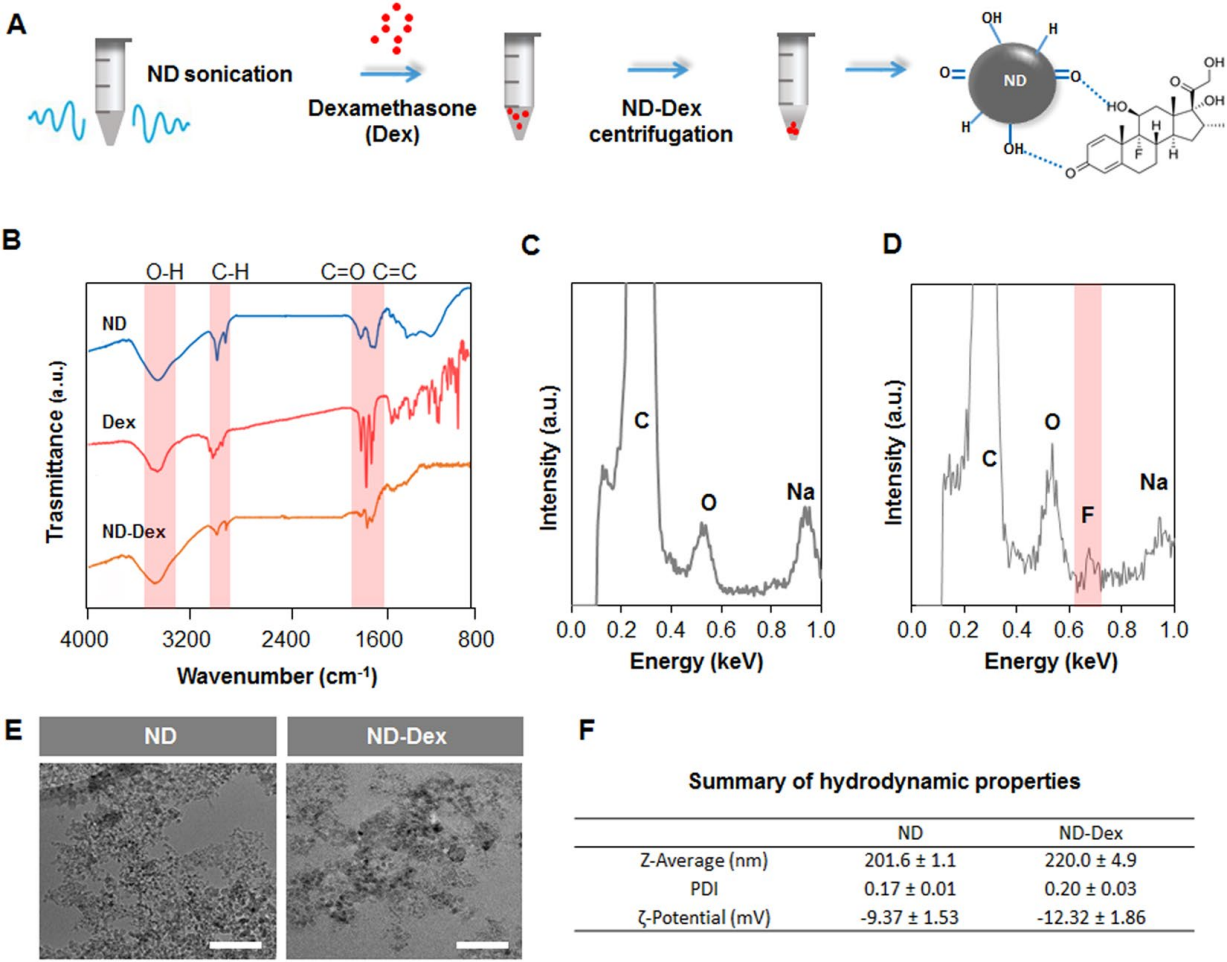
Preparation and characterization of the ND-Dex complex. (**A**) Schematic describing the steps necessary to prepare the ND-Dex complex and the possible interactions between the drug and the ND surface. (**B**) FT-IR spectra in the range of 4000 to 800 cm^−1^ of ND, Dex, and the complex. Highlighted in red are the characteristics FT-IR peaks defining the main functional groups involved in the complex formation. (**C**) The EDX spectra of ND showed the presence mainly of carbon and oxygen on the NDs surface. (**D**) EDX spectra of the ND-Dex conjugate indicating the presence of fluorine on the surface of ND, thus confirming the adsorption of Dex on the NDs surface (**E**) TEM images of ND and ND-Dex complex suspensions in water (Scale bar = 50 nm). (**F**) Comparison of the hydrodynamic properties of ND and the ND-Dex complex in ultrapure water. Data are expressed as mean ± S.D. (n = 3).

The first part of our investigation focuses on successfully binding the model osteoinductive drug Dex to the surface of NDs in order to design a nanocomplex with osteogenic properties. NDs have previously been shown to adsorb poorly water soluble drugs on their surface through several theorized mechanisms, such as electrostatic interactions and hydrogen bonding with polar functional groups^31^. The adsorption of Dex on the surface of NDs was obtained following the procedure described in (Fig. 1A) and was confirmed through Fourier transform infrared spectroscopy (FTIR) and Energy dispersive X-Ray spectroscopy (EDX). FTIR of NDs revealed the presence of hydroxyl (3421 cm^−1^, ν O-H), alkyl (2917 cm^−1^, ν C-H), and carbonyl groups (1714 cm^−1^, ν C=O) which can establish hydrogen bonds and dipole-dipole interactions with Dex. After centrifugation of a 10:1 ND:Dex (w/w) suspension, FTIR analysis of the resulting pellet displayed the characteristic peaks of Dex (1705 cm^−1^, ν C=O; 1658 cm^−1^ and 1618 cm^−1^, ν C = C) in the complex (Fig. 1B). Furthermore, the EDX analysis of NDs (Fig. 1C) and the ND-Dex system (Fig. 1D) showed the presence of fluorine which was absent in the EDX spectra of NDs, thus confirming the presence of Dex. In both cases, a well dispersed ND suspension (5–10 nm) in diameter and of irregular shape was observed using TEM microscopy (Fig. 1E). Dynamic light scattering (DLS) measurements of NDs indicated a bigger hydrodynamic diameter compared to their original size. This result can be related to the natural colloidal stability of nanomaterials in water which causes them to aggregate when the ζ-potential is generally less than ±30 mV^47^. DLS of the ND-Dex displayed an increase in the hydrodynamic diameter (Fig. 1F) which suggests the presence of the drug on their surface. This hypothesis was further confirmed by the reduction of the value of the ζ-potential from approximately −9.4 to −12.3 mV for the ND-Dex complex. This decrease in the ζ-potential can be mainly attributed to the presence of a higher amount of oxygen-containing functional groups such as carbonyl and hydroxyl groups (C=O, -OH) on the NDs’ surface^22^. Finally, it was important to determine the quantity of Dex effectively adsorbed on the surface of the NDs after centrifugation. Because of the broad UV absorbance of NDs which overlaps with the UV profile of Dex, dexamethasone fluorescein isothiocyanate (Dex-FITC) was used for the quantitative binding studies. Visible spectra analysis of the ND-Dex-FITC complex in a 10:1 ratio pre- and post-centrifugation allowed the quantification of the percentage of Dex-FITC adsorbed on the NDs’ surface which was around 65 ± 3% of the original quantity. Representative visible spectra of ND-Dex-FITC in the range of 400–600 nm revealed a decrease in the absorbance values after centrifugation due to the physisorption of Dex-FITC on the ND’s surface. As expected, no change was detected in the absorbance values after spinning the Dex-FITC solution in the absence of NDs (Figure S1). Overall these results confirm the successful formation of the ND-Dex complex and are consistent with previous literature^48^.

### Retention of bioactivity of Dex upon complexation with NDs

**Figure 2:**
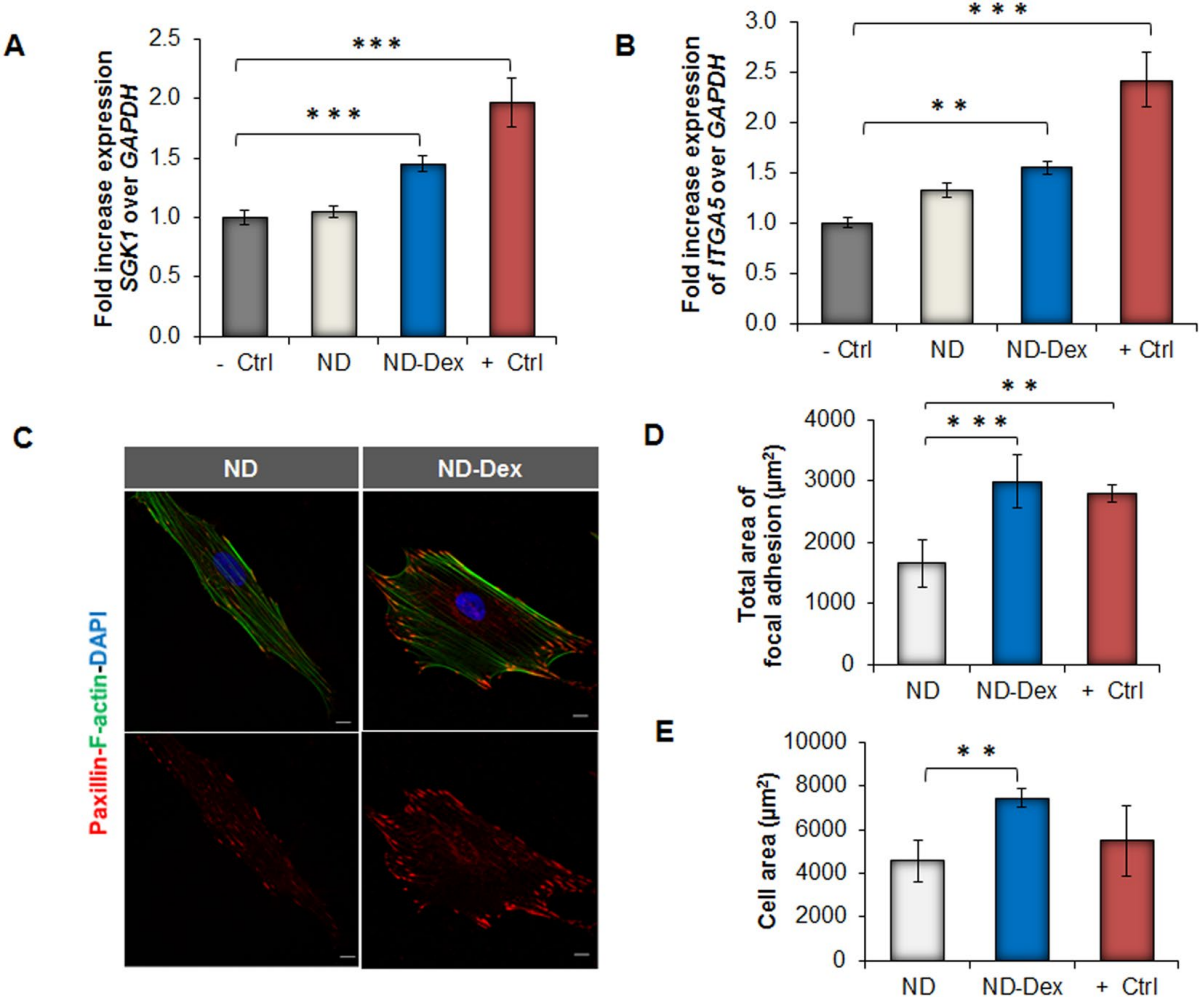
Influence of ND-Dex complex on hASCs gene and focal adhesion expression. (**A**) Relative expression of *SGK1* after hASCs were exposed for one hour to the ND-Dex complex (ND-Dex). Other groups shown, from left to right: untreated hASCs (−Ctrl), hASCs exposed to 25 µg/mL ND suspension (ND), and the drug only (+Ctrl). (**B**) Relative expression of the cytoskeletal element integrin α-5 (*ITGA5*) after six hours of exposure following the same treatment and using the same experimental groups as described previously. (**C**) Fluorescence immunostaining of Paxillin of hASCs after exposure to 25 µg/mL ND suspension and to 25 µg/mL of the ND-Dex complex for 24 hours. (Scale bar = 10 µm). Top images display the combined cell staining including Actin (green) and DAPI (blue) while bottom images represent Paxillin (red) pictures. (**D**) Quantification of the total area of focal adhesion using ImageJ. Results are reported as mean ± S.D., (n = 5). The group (+Ctrl) refers to hASCs exposed to unmodified Dex. (**E**) Quantification of the cell surface area using ImageJ after exposure either to NDs or to the ND complex for 24 hours. Results are reported as mean ± S.D., (n = 5). (*p < 0.05, **p < 0.01, ***p < 0.001).

As the next step of our study, we investigated whether the ND-Dex complex was able to regulate gene and focal adhesion expression of hASCs which represents an indirect confirmation that Dex remained bioactive once complexed with NDs. We first investigated the serum/glucocorticoid-regulated kinase 1 (*SGK1*), a genetic marker upregulated upon Dex exposure^49^. *SGK1* is reported to activate osteogenic differentiation by regulating the stability of microtubules and increasing the number of focal adhesion sites in stem cells^49^. After treatment for one hour with the ND-Dex complex, hASCs showed an increased expression of *SGK1* compared to the other groups (Fig. 2A). We then analyzed the expression of integrin α5 (*ITGA5*) which is another marker activated by Dex that is also associated with the osteogenic differentiation of mesenchymal stem cells^50^. Similarly to *SGK1*, the expression of *ITGA5* significantly increased in hASCs incubated with ND-Dex for six hours (Fig. 2B). In addition to the changes in gene response, we studied the expression of Paxillin (PXN), a protein that modulates focal adhesion activity by binding to β-integrin cytoplasmic domains in the presence of Dex. PXN immunostaining revealed that the treatment of hASCs with ND-Dex resulted in increased focal adhesion surface area with respect to the control group of unmodified NDs (Fig. 2C). Quantification of both focal adhesion and cell area confirmed that the bioactivity of Dex was retained after complexation with NDs (Fig. 2D,E). These results demonstrated that the ND-Dex was able to modulate the gene and focal adhesion expression of hASCs. The integration of this complex in a GelMA hydrogel was the next stage of our investigation in designing a bioactive scaffold that induces osteogenic differentiation of hASCs.

### Confirmation of NDs ***in vitro*** and ***in vivo*** biocompatibility

**Figure 3:**
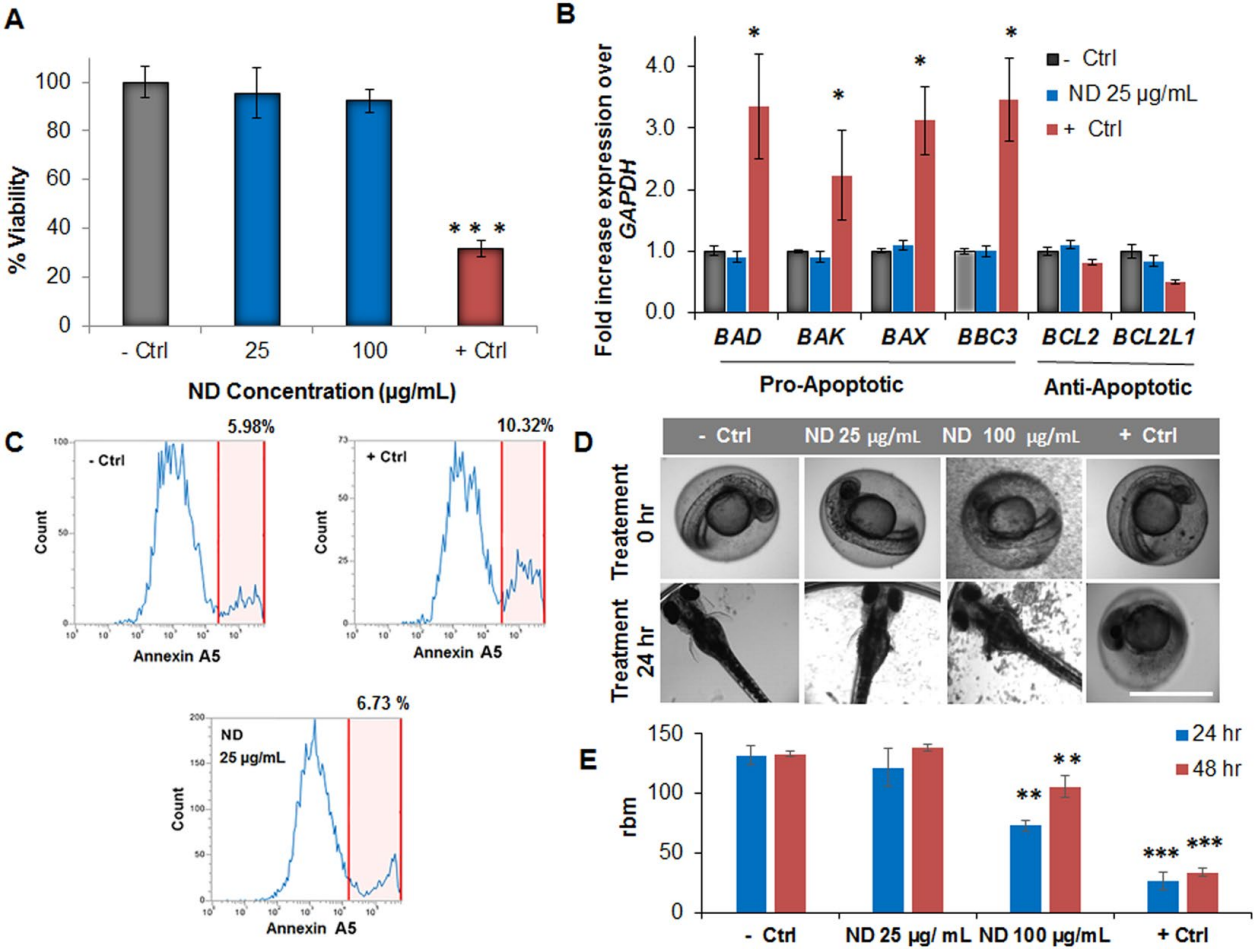
Biocompatibility studies of NDs. (**A**) MTS assay on hASCs exposed to varying concentrations of NDs for 12 hours. NDs showed no significant toxicity up to 100 µg/mL. Results are reported as mean ± S.D, (n = 3). (**B**) qPCR analysis of different pro-apoptotic and anti-apoptotic markers. hASCs showed no increase in apoptotic marker expression after exposure to NDs (25 µg/mL) for 12 hours. The reported groups are respectively untreated hASCs (−Ctrl), cells exposed to NDs (ND), and the hASCs exposed to the drug camptothecin 50 nM (+Ctrl). Results are shown as mean ± S.D, (n = 3). (**C**) FACS analysis of hASCs stained for Annexin A5-Alexa Fluor 488 to detect the presence of apoptotic cells (highlighted in red) after 24 hours exposure to NDs. A comparable % of apoptotic cells were found in the ND group compared to the untreated hASCs. (**D**) Bright field images of zebrafish embryos showing the process of hatching after 24 hours of treatment with two different concentration of NDs (Scale bar = 1 mm). (**E**) Heartbeat per minute of zebrafish embryos after 24 and 48 hours of treatment. Results are indicated as mean ± S.D, (n = 3). (*p < 0.05, **p < 0.01, ***p < 0.001).

Before moving ahead with the design of our 3D nanocomposite scaffold, we first evaluated the biocompatibility of NDs to confirm that they do not induce any cytotoxic effects when used as a nanocarrier of Dex. We tested the viability of hASCs using an MTS assay by testing different concentrations of NDs up to 100 µg/mL and observed no significant reduction in cell viability after 12 hours (Fig. 3A). Furthermore, we tested the gene expression of hASCs for a variety of pro-apoptotic (*BAD, BAX, BAK, BBC3*) and anti-apoptotic genes (*BCL2, BCL2L1*) which are widely reported to be associated with mitochondria-related apoptosis. qPCR analysis revealed no difference in the expression of these genes when hASCs were exposed to NDs for 12 hours (Fig. 3B). Moreover, to support our qPCR findings, Fluorescence-activated cell sorting (FACS) analysis was carried out to measure the percentage of apoptotic cells as a result of exposure to NDs. hASCs were stained for the presence of Annexin A5 which is used to detect cells that have expressed phosphatidylserine on their surfaces during apoptosis. hASCs treated with NDs for 24 hours had similar percentage of apoptotic cells with respect to the negative control group (Fig. 3C). Finally, to assess the viability *in vivo*, we tested the biocompatibility of NDs using a zebrafish embryo model which is a well-known strategy to investigate the toxicity of nanomaterials^51^. At all tested concentrations, NDs did not cause any significant decrease in the hatching rate which was found to be 75% in all groups tested. Also, no detectable change in embryo morphogenesis or decrease in heart rate was observed after 24 and 48 hours of exposure except at the highest tested ND concentration (Fig. 3D,E). Heart beating videos of zebrafish for the different groups after 48 hours of treatment are available in the supplementary info. (Movies S1–S4).

### Effect of NDs on the physical and mechanical properties of GelMA scaffold

**Figure 4:**
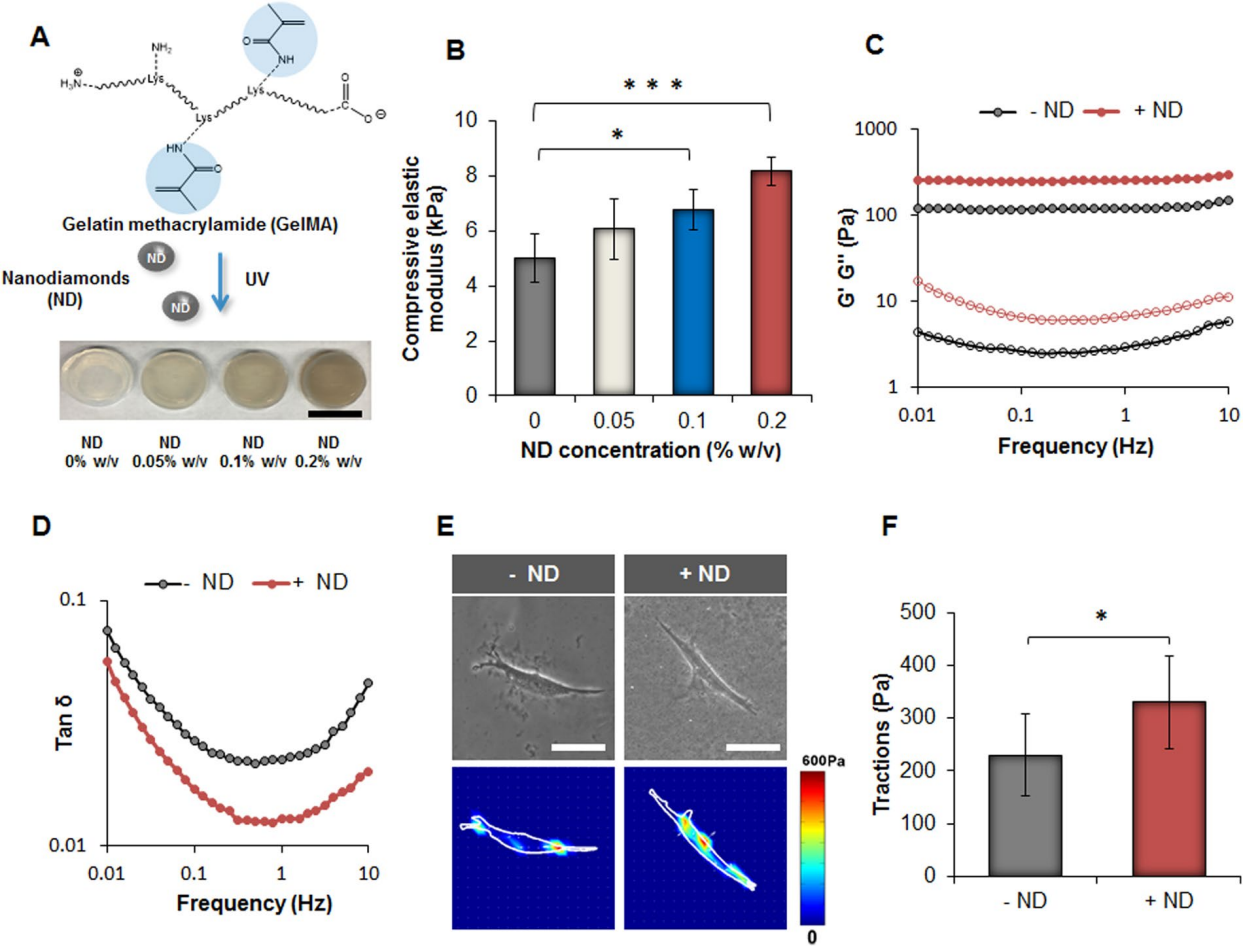
Influence of the NDs on the mechanical properties of GelMA hydrogels and evaluation of the substrate stiffness on hASCs traction forces. (**A**) Schematic indicating the functional groups in GelMA involved in the process of crosslinking using UV irradiation. On the bottom, an image displaying the different GelMA nanocomposite hydrogels prepared using several concentrations of NDs. (Scale bar = 15 mm). (**B**) Compressive elastic modulus of the different samples obtained by varying the concentration of NDs. Results are reported as mean ± S.D., (n = 5). (**C**) Frequency sweep carried out in the range of 0.01 up to 10 Hz indicates an increase in the value of the storage modulus (G’) for the system containing NDs (0.2% w/w). (**D**) Tan δ (G”/G’) profiles indicate a decrease of this parameter in the presence of NDs (0.2% w/w) in all the range of frequencies tested. (**E**) Phase contrast pictures (scale bar = 200 µm) and traction map images of hASCs cultured on GelMA hydrogels (−ND) and nanocomposite hydrogels (+ND) after 24 hours. (**F**) Root-mean-square (RMS) traction values of hASCs indicating an increase in cell traction on the substrate containing NDs. Results are showed as mean ± S.D., (n = 8). (*p < 0.05, ***p < 0.001).

After the evaluation of the biocompatibility of NDs, it was necessary to study how NDs influenced the physical and mechanical properties of GelMA scaffold. GelMA is a methacrylamide derivate of gelatin which facilitates the preparation of photochemical hydrogels that present excellent biocompatibility associated with tunable physical properties according to their degree of methacrylation^46^. GelMA is a versatile polymer that has been previously combined with other carbon nanomaterials such as graphene oxide (GO) or carbon nanotubes (CNT) in cardiac tissue engineering applications^19, 52^. In this work, we used a similar strategy by combining NDs with GelMA to design a novel nanocomposite biomaterial that could serve as a cell scaffold capable of modulating osteogenic differentiation of hASCs. Several GelMA/ND nanocomposites were fabricated by incrementally varying the concentration of NDs from 0.05 to 0.2% w/v and keeping the amount of GelMA constant at 7% w/v (Fig. 4A). SEM images of both GelMA and GelMA/ND 0.2% w/v nanocomposite hydrogels revealed the presence of a similar interconnected porous network (Figure S2A). The swelling ratio was similar among the groups tested (Figure S2B) and the observed result could be attributed to the absence of ionic groups on the surface of NDs. On the contrary, the compressive elastic modulus steadily increased with the addition of NDs to the GelMA network. The GelMA/ND hydrogel with an ND concentration of 0.2% w/v demonstrated a compressive modulus nearly double that of GelMA alone (Fig. 4B). 0.2% w/v was chosen as the upper limit for the range of ND concentrations used to design the nanocomposite hydrogels because no significant changes in stiffness were observed with a higher amount of NDs. Although stress-strain curve profiles of both systems presented a different initial slope, they each exhibited the same strain at the point of fracture. The ultimate fracture compressive strength was nearly double (~260 kPa vs. ~180 kPa) in the nanocomposite system containing NDs 0.2% w/v (Figure S2C).

To further confirm the increase in stiffness provided by the presence of NDs in the GelMA polymeric network we carried out a frequency sweep in the viscoelastic region defined by preliminary strain sweep studies. Two important parameter that can be obtained from a frequency sweep are the values of the storage modulus G′, and the loss modulus G″, which are representative of the elastic and the viscous properties of a hydrogel. Mechanical spectra of the GelMA/ND 0.2% w/v showed higher values of G′ in all frequencies tested. Moreover in both cases, G′ remained the same irrespective of the frequency applied which is a typical behavior of solid-like materials (Fig. 4C). The corresponding tan(δ) profiles revealed useful information regarding the effect of NDs in tuning the mechanical properties of GelMA scaffolds. Specifically, the observed decrease in tan(δ) in the presence of NDs in all frequencies tested indicates that the nanomaterial could interact with the polymeric network by acting as an additional crosslinker (Fig. 4D). The mechanism behind this reinforcement could be attributed to the presence of hydroxyl and carbonyl groups on the surface of NDs that can form hydrogen and dipole interactions with the polymeric network in addition to covalent bonds during the process of radical photopolymerization. These results provide conclusive evidence that NDs are capable of improving the mechanical properties of GelMA scaffolds in the same manner as other carbon nanomaterials as reported in previous studies^53^. The improvement of mechanical properties as a result of the addition of NDs also suggests a possible explanation for the different hydrogel degradation profiles observed between GelMA and GelMA/ND hydrogels (Figure S2D). GelMA networks degraded completely in a solution of collagenase 0.5 U/mL after five days compared to the GelMA/ND 0.2% w/v system which lost only 40% of its original weight in the same time frame. The delayed degradation further confirms that the inclusion of NDs can reinforce the hydrogel network and can hinder the process of enzymatic degradation.

### Effect of ND on stem cell traction

We also investigated how the increased substrate stiffness in the nanocomposite system could affect the behavior of hASCs in terms of the traction forces exerted on the gels’ surface **(**Fig. 4E). It is well established that substrate rigidity can profoundly influence cellular responses^54^. For instance, the design of a substrate with higher stiffness allows for an increase in cell-matrix interactions which can encourage stem cell differentiation to proceed towards an osteogenic lineage^55, 56^. According to our results, hASCs displayed higher traction forces on the surface of the nanocomposite system compared to the GelMA control as a response to a more rigid surface (Fig. 4F). These results indirectly confirm the change in the hydrogel mechanical properties caused by the presence of NDs in the GelMA network. However, further investigation is necessary to evaluate whether the potential increase in substrate stiffness observed in the nanocomposite system can promote differentiation of hASCs towards an osteogenic lineage in 2D culture.

### GelMA-ND for micropatterns and bioprinting applications

**Figure 5:**
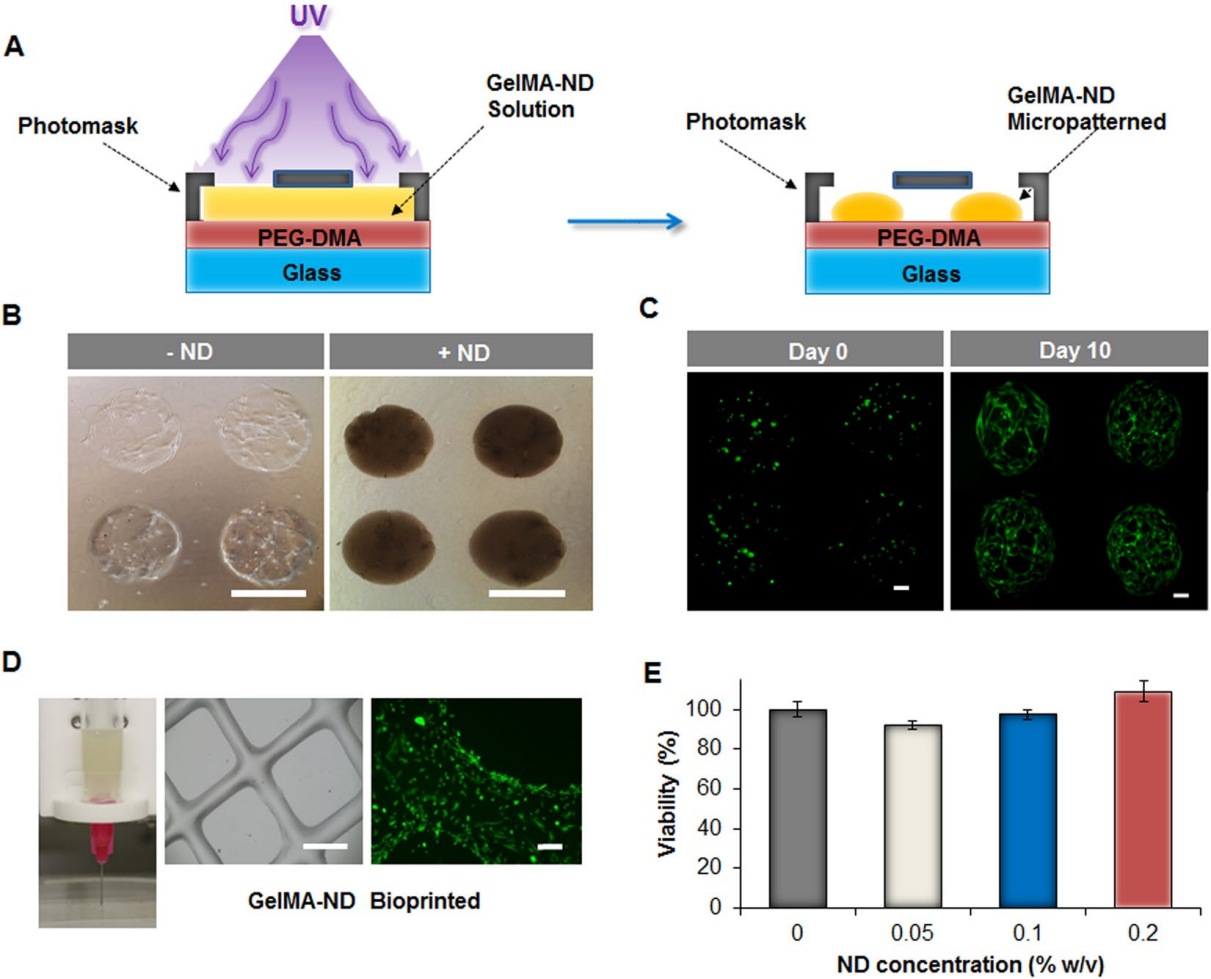
Microfabricated structures using GelMA/ND nanocomposite hydrogels and evaluation of their biocompatibility. (**A**) Schematic describing the process of micropatterning of the GelMA/ND prepolymer solution into nanocomposite hydrogel with a 1 mm diameter. (**B**) Brightfield images of 1 mm in diameter micropatterned hydrogels with (+ND) and without (−ND) NDs encapsulating hASCs in 3D after ten days of culture. (Scale bar = 1 mm). (**C**) Calcein-AM staining of hASCs cultured in GelMA/ND micropatterned hydrogels at different time points. hASCs proliferated and spread throughout the microfabricated constructs after ten days. (Each scale bar = 200 µm). (**D**) The prepolymer GelMA/ND solution can also be bioprinted into more complex structures as shown in the phase contrast image (scale bar = 1 mm). The calcein-AM staining (green) shows the hASCs that were seeded on the bioprinted nanocomposite GelMA/ND and cultured for five days. (Scale bar = 100 µm). (**E**) MTS assay of hASCs encapsulated in GelMA/ND nanocomposite hydrogels demonstrated excellent biocompatibility regardless of the ND concentration in the gel. Results are reported as mean ± S.D., (n = 5). No statistical difference was observed among the groups tested.

To further investigate the ability of NDs to enhance the mechanical properties of GelMA hydrogels, we studied the microscale properties of this novel GelMA-ND scaffold by microfabricating constructs following several strategies as shown in Fig. 5. In the first method, which utilized photolithographic techniques, photomasks were used to create micropatterned GelMA/ND cell scaffolds (Fig. 5A,B). hASCs were able to proliferate and migrate throughout the 1 mm diameter cylindrical hydrogel constructs after 10 days of culture (Fig. 5C). In the subsequent strategy, the GelMA/ND mixture was bioprinted into defined geometries and shapes (Fig. 5D) to form scaffolds that showed excellent biocompatibility regardless of the concentration of NDs used for their preparation (Fig. 5E).

### Gel-MA nanocomposite hydrogel as strategy to modulate the release of Dex

**Figure 6:**
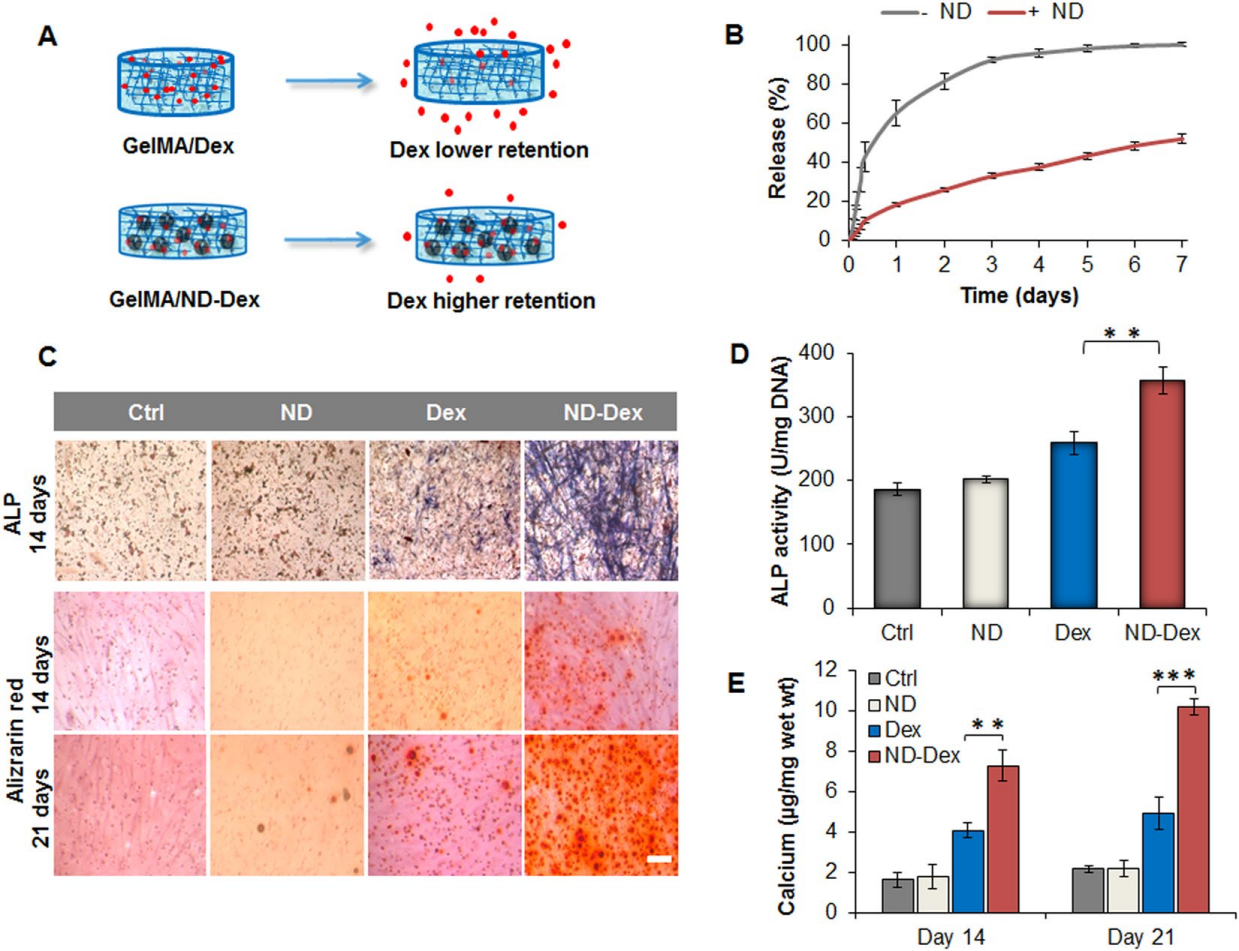
Modulation of dexamethasone release using GelMA/ND nanocomposite hydrogels and assessment of osteogenic differentiation of hASCs. (**A**) Schematic representing the different diffusion rate of dexamethasone with higher retention of the drug in the nanocomposite hydrogels compared to the scaffold without NDs. (**B**) Comparative release profiles of dexamethasone-Fluorescein isothiocyanate (Dex-FITC) from GelMA hydrogels and the nanocomposite scaffold over a week. Results are indicated as mean ± S.D., (n = 4). (**C**) In the first row, images are displaying alkaline phosphatase (ALP) staining of hASCs encapsulated in GelMA hydrogels after 14 days of culture in osteoconductive media. In the second and third-row, alizarin red staining of hASCs at 14 and 21 days of culture in osteoconductive media. (Scale bar = 100 µm). (**D**) ALP quantification of hASCs after 14 days of culture in osteoconductive media showing a significant increase in ALP expression for the nanocomposite system compared to the control groups. Results are reported as mean ± S.D., (n = 3). (**E**) Calcium content quantification after 14 and 21 days of hASC differentiation induced by osteoconductive media. Results are shown as mean ± S.D., (n = 3). Groups tested are respectively GelMA hydrogels (Ctrl), GelMA/ND 0.2% w/v nanocomposite scaffold without Dex (ND), GelMA hydrogels containing the free drug (Dex) and the nanocomposite system containing the complex (ND/Dex). (**p < 0.01, ***p < 0.001).

In the final step of our study, we wanted to verify whether the presence of the bioactive ND-Dex complex within GelMA scaffolds represented a successful strategy in controlling the osteogenic differentiation of hASCs. One of the disadvantages of conventional hydrogels, including GelMA, is that they do not provide any control over the release of the cargo loaded^57^. To overcome this downfall, we investigated whether or not the ND-Dex complex would enable a higher retention of Dex within the polymeric network of GelMA (Fig. 6A). We hypothesized that an increased retention of Dex within the GelMA scaffold would enhance the differentiation of hASCs encapsulated in 3D. We first investigated the release profile of Dex-FITC as free drug loaded in a GelMA scaffold in comparison with the nanocomposite GelMA/ND 0.2% w/v formulated with the ND-Dex-FITC complex. Dex-FITC instead of Dex was chosen for the study in order to avoid any interference caused by the presence of the photoinitiator Irgarcure 2959 which absorb in the UV region as Dex. According to the results, the amount of Dex-FITC released from the nanocomposite hydrogels was significantly extended compared to GelMA controls (Fig. 6B). At 72 hours, around 50% of Dex-FITC remained in the nanocomposite hydrogel compared to less than 10% in GelMA scaffold. The release profiles (Figure S3) for each group were fit to a first order Fickian diffusion model as well as the Korsmeyer-Peppas model^58^. The Korsmeyer-Peppas model is a semi-empirical model with a release exponent parameter (n) that indicates the release behavior of the system. When n < 0.5 the release follows Fickian diffusion, but if 0.5 < n < 1 then the diffusion is based on non-Fickian mechanisms. The experimental release data of Dex-FITC from GelMA matched a first order diffusion model, while the release profile from the GelMA-ND hydrogels did not follow the first order kinetics. For this reason, the results were fit to the Korsmeyer-Peppas model (n = 0.578 ± 0.053) to elucidate the deviation from the first order kinetics. All of the parameters for the two systems are summarized in Table S1. This difference can be related to the possible interactions between Dex-FITC and NDs including dipole-dipole and hydrogen bonding that can be responsible for the non-Fickian mechanism observed in the GelMA-ND system.

### Modulation of the osteogenic properties of GelMA scaffolds using ND-Dex complex

After confirming the enhanced retention of Dex in the nanocomposite hydrogels, we investigated its potential to initiate osteogenic differentiation of encapsulated hASCs. The nanocomposite hydrogels containing ND-Dex promoted enhanced differentiation compared to all controls as measured by alkaline phosphatase (ALP) and alizarin red staining (Fig. 6C).

Both quantitative and qualitative evaluations of ALP activity were performed at 14 days. ALP activity was significantly higher in the nanocomposite hydrogel containing the ND-Dex complex compared to that of GelMA containing the same concentration of free Dex (Fig. 6D). Alizarin red colorimetric staining allows qualitative comparison of bone-like calcium deposits. At both 14 and 21 days, all control groups showed minor mineralization, while ND-Dex displayed a significantly greater extent of calcium deposition (Fig. 6E). Similarly, immunostaining for osteocalcin (OCN) at day 21 provided further evidence of the successful hASCs’ osteogenic differentiation. OCN is an osteoblast-specific marker that serves both endocrine and mineralization functions. ND-Dex nanocomposites showed increased OCN staining, while the control groups displayed very little or no staining (Figure S4).

**Figure 7:**
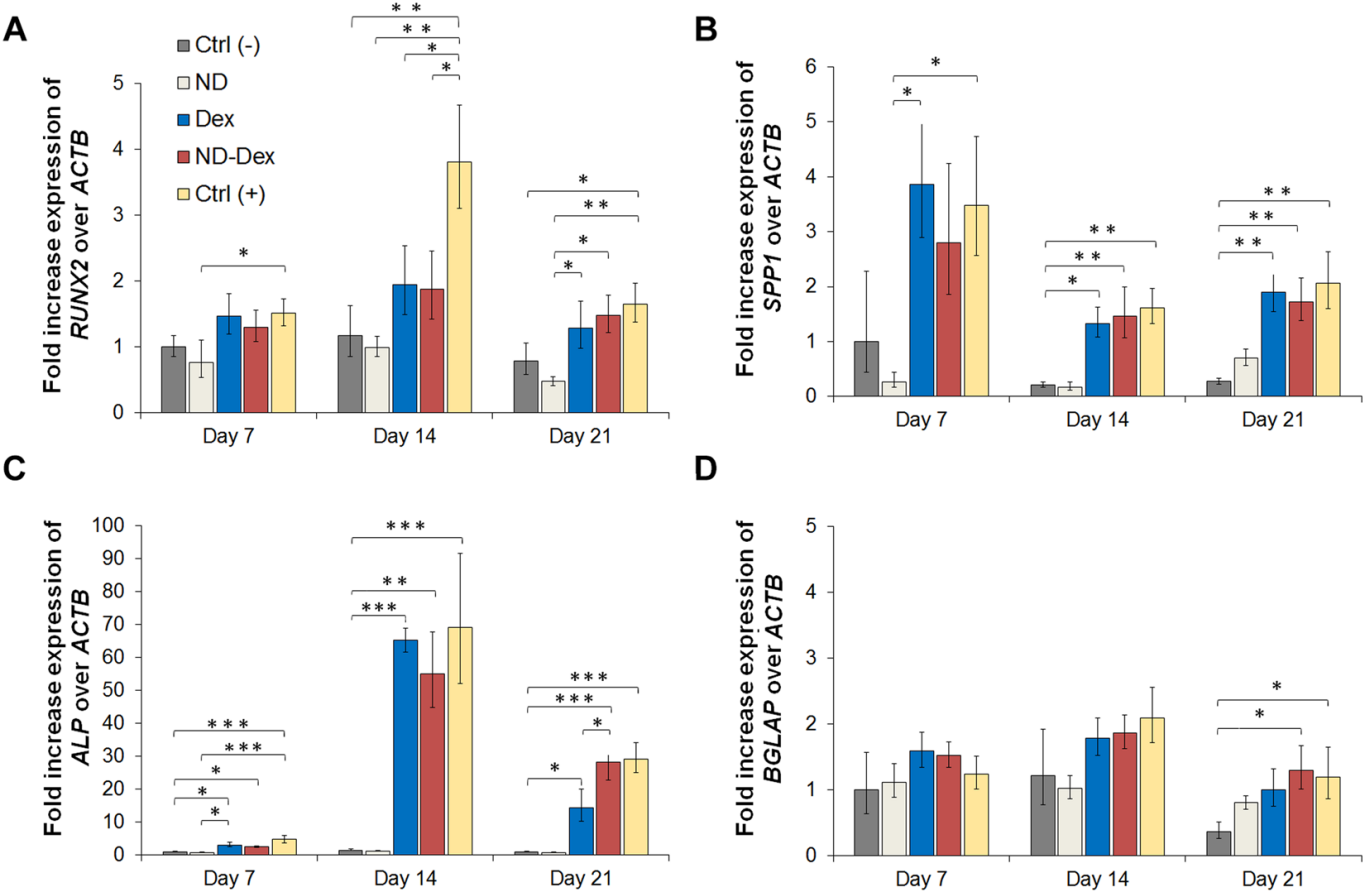
Assessment of osteogenic differentiation of hASCs by qPCR analysis. The fold expression increase of different genes have been evaluated including (**A**) *RUNX2*, (**B**) osteopontin *SPP1*, (**C**) alkaline phosphatase *ALP* and (**D**) osteocalcin *BGLAP*. β-actin *ACTB* has been used as control gene and data were calculated following the relative method ΔΔCt. Results are normalized based on the values obtained from the Ctrl (−) group at day 7. Groups tested are respectively hASCs cultured using GelMA hydrogels in basal media Ctrl (−), GelMA/ND 0.2% w/v nanocomposite scaffold without Dex in basal media (ND), GelMA hydrogels containing dexamethasone 1 µM in osteoconductive media (Dex), the nanocomposite system containing the complex in osteoconductive media (ND/Dex) and hASCs cultured in osteoinductive media Ctrl (+). Results are shown as mean ± S.D. (n = 3) (*p < 0.05 **p < 0.01, ***p < 0.001).

Finally, qPCR analysis confirmed the differentiation of hASCs carried out for 21 days in osteoconductive media for the Dex and the ND-Dex groups when compared to the negative controls. However, no significant difference was found in the gene expression of *RUNX2* and *SPP1* in the Dex and ND-Dex groups after 14 and 21 days of differentiation, which were both activated to the same extent with respect to the positive control **(**Fig. 7A,B **)**. On the contrary, the fold increase expression of *ALP* was significantly higher in the ND-Dex (28.14 ± 6.6) group after 21 days of culture compared to the Dex samples (14.3 ± 5.7) **(**Fig. 7C **)**. Similarly, only the ND-Dex group and the positive control showed a significant increase in the expression of the later osteogenic marker *BGLAP* after 21 days compared to the negative controls, while no significant change of *BGLAP* expression was found in the Dex group. **(**Fig. 7D **)**. Both findings can be explained by the higher retention of the drug in the ND-Dex group, which was still present in the nanocomposite hydrogel after 21 days of culture.

## Conclusions

We successfully engineered a novel nanocomposite hydrogel composed of GelMA and nanodiamonds as a promising scaffold to control osteogenic differentiation of hASCs. NDs are a versatile and biocompatible platform that can serve as both as a carrier of drugs and as a nanofiller agent to modulate the mechanical properties of scaffolds. NDs enhanced the mechanical properties of GelMA hydrogels as demonstrated by compressive and rheological studies and indirectly by evaluating the traction forces of hASCs on hydrogels of varying stiffness. Furthermore, the adsorption of Dex onto the surface of NDs enabled a sustained release of Dex in comparison with conventional GelMA scaffolds that were not able to retain Dex over time. Finally, the inclusion of the ND-Dex complex enhanced osteogenic differentiation of encapsulated hASCs in 3D. Overall, these findings highlight the benefits of incorporating NDs in the design of gelatin-based nanocomposite scaffolds and how their unique properties can be harnessed to develop new bone tissue engineering strategies specifically for the treatment of non-load bearing defects.

## Methods

### ND-Dex complex preparation

Detonation NDs (Nanostructured & Amorphous Materials, Inc., USA) were used without any further purification from the company. NDs were dispersed in ethanol (0.2% w/v) and sonicated for 30 minutes (20 kHz, 2 sec on, 1 sec off) with an Ultrasonic Processor 500 W Ultrasonic, 20 kHz (Midwest Scientific, Inc, USA). Subsequently, NDs were mixed with a solution of Dex (Sigma-Aldrich, USA) (0.02% w/v) prepared in ethanol (Fischer, USA). Then, the mixture was centrifuged for 20 minutes at 14,000 rpm and the supernatant discarded. The pellet was either resuspended in water or dried for further characterization. To quantify the amount of drug adsorbed on the surface of the NDs, the preparation of the complex was obtained using Dex-FITC (Sigma-Aldrich, USA) due to the interference of NDs in the UV region. Visible spectra in the range of 400–600 cm^−1^ were recorded pre- and post-centrifugation and the absorbance of Dex-FITC at 490 nm was used to determine the amount of drug remaining in solution after the centrifugation of the complex.

### Characterization of NDs and ND-Dex complex

The morphology and size of the NDs and ND-Dex were investigated by transmission electron microscopy (TEM). The TEM samples were prepared by immersing the carbon-coated 200-mesh copper grids into a diluted suspension of NDs (0.25 µg/mL) in deionized water followed by washing the grids with deionized water and drying in a desiccator for few hours. The same procedure was followed for the ND-Dex samples obtained resuspended in water the pellet obtained as previously described. The dried grids were analyzed, and high-resolution TEM images were recorded (FEI, Tecnai F20XT, USA). The hydrodynamic diameter and the zeta potential of the NDs after sonication for 30 minutes (20 kHz, 2 sec on, 1 sec off) were measured on a ZetaPALS zeta potential analyzer (Brookhaven Instruments Corporation, USA) by hydrodynamic light scattering and laser Doppler electrophoresis. Finally, surface chemistry was characterized by energy-dispersive X-ray spectroscopy (EDX), and Fourier transform infrared spectra (FT-IR) using a Bruker Vector-22 FTIR spectrophotometer (PIKE Technologies, USA). For FT-IR spectra analysis, detonation ND or the dried ND-Dex pellet were mixed in KBr (Fischer, USA) and spectra were recorded in the range of 400 up to 4000 cm^−1^ with a resolution of 1 cm^−1^.

### Biocompatibility studies of NDs

Human adipose-derived stem cells (hASCs) (RoosterBio, USA) were maintained in Invitrogen alpha-Minimum Essential Medium (α-MEM) with 15% fetal bovine serum (FBS) (Thermo-Fischer Scientific, USA) and 1% penicillin/streptomycin (Thermo-Fischer Scientific, USA) at 37 °C and 5% CO_2_. Passages 3–5 were used for all the studies. hASCs were exposed to different concentrations of NDs in phosphate buffer saline PBS (25 and 100 µg/mL) for 24 hours followed by MTS assay (Promega, USA) to test the biocompatibility of NDs. As a positive control, camptothecin at the concentration of 50 µM was added to the media to induce apoptosis. Apoptotic gene expression of hASCs after treatment with NDs was also investigated using RT-qPCR analysis. mRNA from each group was first extracted using an RNeasy Mini Kit (Qiagen, Germany). mRNA samples were measured using a NanoDrop (Thermo Scientific, USA) and converted to cDNA using the High-Capacity cDNA Conversion Kit (Applied Biosystems, USA). Finally, gene expression was evaluated using a mixture of predesigned primers and the KiCqStart SYBR Green Master Mix (Sigma-Aldrich, USA). All qPCR reactions were carried out with a Mastercycler Realplex (Eppendorf, Germany) and the fold expression levels were calculated using the relative ∆∆Ct method, using *GAPDH* as the housekeeping gene. To further confirm the NDs’ biocompatibility, FACS analysis was carried out to detect the presence of apoptotic cells after treatment with NDs for 24 hours. hASCs without any treatment and the ones exposed to the drug camptothecin (50 µM) were tested as negative and positive control respectively. Briefly, hASCs were trypsinized and washed twice with cold PBS. Subsequently, the Annexin A5 binding buffer was used to resuspend hASCs (1 × 10^6^ cells/ml). 10 µl of Annexin A5-FITC was included to the resuspended hASCs and incubated for 30 min in the dark. Following incubation, 5 µl of propidium iodide (PI) was added to the resuspended hASCs and incubated for 10 min in the dark. The apoptosis ratio of hASCs was then immediately measured using the Attune NxT flow cytometer (Thermo-Fischer Scientific, USA).

Finally, as an *in vivo* model, the same concentrations of NDs (25 and 100 µg/mL) were tested on a zebrafish embryo model. Embryos were cultured in zebrafish embryo buffer (5 mM NaCl, 0.17 mM KCl, 0.33 mM CaCl2, 0.33 mM MgSO4; pH 7.4)^59^ and exposed to NDs in the same medium 24 h post fertilization. Embryos were observed daily to detect any alterations in morphology and to determine their hatching rate. Heartbeat rate was monitored using a brightfield microscope EVOS cell imaging system (Thermo-Fischer Scientific, USA), at 24 and 48 hours after treatment. Results were compared to the negative and positive controls groups respectively embryos not treated and exposed to camptothecin 100 nM.

### Bioactivity of the ND-Dex complex

hASCs were seeded into chamber slides, allowed to grow in complete media, and then serum starved for 48 hours. Subsequently, hASCs were treated with the ND-Dex complex (25 µg/mL) containing an amount of Dex equal to 1 µM, and as control groups, cells were exposed only to NDs (25 µg/mL) and only to Dex (1 µM). Gene expression for Dex-related markers in the different groups was then evaluated at 1 and 6 hours post-treatment using the qPCR procedure as previously described. Cells were then fixed in 4% paraformaldehyde for 5 minutes at 37 °C and permeabilized with 0.1% Triton-X100 for 10 minutes at room temperature. Samples were blocked with 5% normal goat serum for 45 minutes at room temperature. Mouse anti-human Paxillin antibody (1:400 with 1% goat serum, Invitrogen, USA) was added to cells and left overnight at 4 °C. Goat anti-mouse AlexaFluor 594 (Invitrogen USA) (1:500 in 1% goat serum) was then added and left for one hour at room temperature. Diamidino-2-phenylindole dilactate (DAPI, Invitrogen, USA) and phalloidin-AlexaFluor488 (Invitrogen, USA) were used to counterstain nuclei and F-actin, respectively. Immunofluorescence images were collected for each sample by laser scanning confocal microscopy (LSCM). All of the immunofluorescence images were processed using ImageJ software, which allowed for quantitation of the total number of adhesion sites and the area of each site.

### GelMA synthesis and nanocomposite hydrogels preparation

Gelatin A (300 bloom grade, from porcine skin) and methacrylic anhydride were purchased from Sigma-Aldrich (USA). GelMA was synthesized as previously described^60^. Briefly, a 10% w/v gelatin solution was prepared in PBS at pH 7.4. Methacrylic anhydride (8 mL) was added dropwise to methacrylate amine groups along the gelatin backbones. The polymeric mixture was stirred vigorously and maintained at 60 °C for two hours after dilution with PBS. The polymeric solution was then transferred to dialysis membranes made of regenerated cellulose (~12–14 kDa cutoff) and dialyzed with deionized water for one week with two daily water changes. The GelMA solution was then frozen at −80 °C and lyophilized for 72 hours.

To fabricate GelMA-ND nanocomposite hydrogels, NDs were sonicated for 30 minutes (20 kHz, 2 sec on, 1 sec off) in deionized water at the concentration of 0.4% w/v prior addition to the GelMA solution in PBS. Nanocomposite hydrogels composition was modified varying the amount of NDs from 0.05% up to 0.2% w/v keeping constant gelatin concentration at 7% w/v and the amount of photoinitiator Irgacure 2959 (Sigma-Aldrich, USA) at 0.1% w/w. Photochemical hydrogels were then obtained after UV irradiation at 350–400 nm (Omnicure S200, Lumen Dynamics, Canada) for 6 minutes at an intensity of 7 mW/cm^2^.

### Physical and mechanical characterization of GelMA-ND nanocomposite hydrogels

Freeze-dried hydrogels were weighed and soaked in PBS at 37 °C. The equilibrium swelling was evaluated by weighing the swollen hydrogel at different time points and the swelling ratio (%) was calculated using the following equation:

1 $$Swelling\,ratio\,( \% )=\frac{Ws-{W}_{d}}{\,Wd\,}\times 100$$

where W_s_ is the weight of the swollen hydrogel, and W_d_ represents the weight of the freeze-dried hydrogel. In addition, the porosity of the hydrogel with and without NDs was evaluated by scanning electron microscopy (SEM). Samples were mounted on a holder with double sided conductive carbon tape and sputter-coated with gold. SEM images were obtained at an acceleration voltage ranging from 1 to 10 kV with an in-lens detector. Finally, for degradation tests, the freeze-dried hydrogels were weighed and then soaked in a collagenase type IV solution (0.5 U/ml) dissolved in PBS (pH 7.4). The degradation tests were performed in an incubator at 37 °C and 5% CO_2_ and the collagenase solution was changed daily. The hydrogels were removed from the collagenase solution at different time points, washed with PBS, and freeze dried. Five samples were used for each time point of the study, and the percentage of degradation was calculated by weight loss using the following equation:

2 $${W}_{L}( \% )=\frac{{W}_{i}-{W}_{f}}{\,\,{W}_{i}\,}\times 100$$

where w_i_ is the initial weight and w_f_ is the weight at different time intervals.

The compressive modulus of the nanocomposite hydrogels was determined using an RSA-III dynamic mechanical analyzer (TA Instruments, New Castle, DE) and evaluated under unconfined uniaxial compression with a 35 N load cell (n = 5). The diameter of the gels was measured using a caliper, while the height was obtained directly using the RSA-III. All mechanical testing were performed on the swollen injectable nanocomposite hydrogels, and the compression probes were lubricated with mineral oil both to minimize gel drying during the test. A compression rate of 0.005 mm/s, corresponding to an average of 15% per min, was used. The compressive elastic modulus (E) was calculated as the slope up to an x-axis value of 10% strain of the stress versus strain curve. Samples were compressed unconstrained to 95% of their original height or to fracture, which was measured directly with the RSA-III.

Finally, frequency sweep in the range from 0.01 up to 10 Hz were recorded for all samples at 37 °C in the viscoelastic region at 1% of strain. Preliminary strain sweep tests were carried out in the range of 0.1 up to 100% of strain to define the range of viscoelastic region. Nanocomposite hydrogels with and without NDs (diameter of 1 cm and thickness of 0.5 cm, n = 3) were tested using a 25 mm serrated steel plate-plate geometry, and a water trap was placed on the geometry to avoid excessive evaporation during the test. Hydrogels were swollen prior the study for 1 hour in PBS pH 7.4, and each study was repeated three times.

### Evaluation of hASCs traction forces on nanocomposite hydrogels

GelMa and GelMA-ND nanocomposite hydrogels were fabricated in combination with fluorescent microbeads (Fisher Scientific, USA) having a diameter of 0.2 µm, to examine the influence of the substrate stiffness on hASCs traction forces on the gels. Microbeads were homogeneously dispersed in the polymeric mixtures by vortexing them with Vortex Mixer 120 V (VWR, USA) for five minutes. Then, each hydrogel was prepared by adding 45 µl of the sample on a petri-dish in between two spacers with a desirable height of 50 µm. Prior irradiation, the drop was flattened between the spacer using a glass slide previously coated with 3-tri(methoxysilyl) propyl methacrylate (TMSPMA) (Sigma, USA). After UV exposure for 60 seconds, the glass slides containing the gels were washed with PBS and soaked in α-MEM containing 15% FBS and 1% penicillin/streptomycin for two hours. hASCs (5,000 cells per gel) were seeded on top of the gels and kept growing for 24 hours. The day after cells were removed from the gel using trypsin and two sets of fluorescent images for the same field of view were taken using an EVOS cell imaging microscope (Thermo-Fischer Scientific, USA) before and after trypsinization. The displacement caused by each cell’s tractional forces was calculated by comparing the fluorescent bead positions between the two sets of images corresponding to the gels in the stress and relax state. The root mean square (RMS) traction was calculated using Matlab (Mathwork Inc.) and obtained from the displacement field, taking into consideration the values of compressive elastic modulus for each hydrogel substrate^61^.

### GelMA-ND nanocomposite hydrogels micropatterning and bioprinting

GelMA-ND micropatterns were created using previously established procedures^62, 63^. Prior to cell encapsulation, glass slides were treated with TMSPMA (Sigma-Aldrich, USA) and coated with a 50 µm layer of polyethylene glycol diacrylate (PEGDA, Mw 1000Da) (Sigma-Aldrich, USA) by UV crosslinking for 60 seconds. To prepare cell-laden micropatterns, hASCs were mixed with a 7% w/v GelMA pre-polymer solution containing 0.2% w/v of ND and 0.1% w/v of the photoinitiator Irgacure 2959 (Sigma-Aldrich, USA). Cells were mixed in the GelMA-ND mixture using a cell density of 1.0 × 10^6^ cells/mL. A 45 µl of pre-polymer solution was then dropped on a petri-dish in between two spacers with a desirable height of 100 µm. Subsequently, the PEGDA coated glass slides were placed on top of the pre-polymeric solution to obtain the thickness above indicated. Finally, a 1 × 1 cm photomask was added on the glass slide and the entire construct was exposed to UV light (7 mW/cm^2^ intensity) for 45 seconds. The PEGDA-coated glass slides containing patterned, crosslinked hydrogel constructs were submerged into warm PBS. Following encapsulation, the cell-laden constructs were cultured in 6-well cell culture plates and supplemented with complete media. hASCs were stained using calcein-AM at 5 and 10 days according to the manufacturer’s protocol (Invitrogen, USA) and images were taken using a fluorescent microscope (Zeiss, Germany).

Finally, a three-dimensional model was designed using a Repetier-Host software and loaded via a computer-aided manufacturing (CAM) software. Constructs were printed using the Inkredible benchtop 3D bioprinter (Cellink, Sweden). Printing was performed at room temperature while keeping the hydrogel dispensing heads at 37 °C. GelMA and GelMA/ND composites were printed using a 25G needle (inner diameter 0.25 mm), an XY-plane speed of 39 mm/s, an extrusion speed of 10 mm/s and a layer thickness of 0.2 mm. Immediately after printing the constructs were UV-crosslinked at 6.9 mW/cm2 for 60 seconds and washed in PBS. The constructs were printed on glass slides treated with TMSPMA and coated with PEGDA. hASCs were then seeded on the bioprinted scaffolds and calcein-AM staining along with MTS assay were carried out to assess cell morphology and viability after 48 hours.

### Release studies of Dex-FITC from GelMA and GelMA-ND nanocomposite hydrogels

Dex-FITC was mixed both as a free drug and as a complex with NDs (10:1) in the GelMA solution (7% w/v) prior to UV irradiation. The final concentration of Dex-FITC included as the free drug in the conventional hydrogel (GelMA) and as a complex with NDs in the nanocomposite system was kept the same (250 µg/mL). Briefly, 40 μL of each system was placed in 96-well plates and crosslinked at 7 mW/cm^2^ for 60 seconds to form cylindrical gels (thickness 1.25 mm) at the bottom of each well. All wells were then filled with 200 μL of PBS and the release was carried out at 37 °C in a shaker incubator setting the rotation speed at 60 rpm. The entire volume was withdrawn every hour for the first 8 hours and subsequently every 24 hours until a plateau was reached. The 96-well plates were protected from the light, and the PBS incubation solutions were measured in a new well plate. The fluorescence intensity of Dex-FITC was detected using a Cytation 5 plate reader (BioTek, USA) working at 493 nm excitation and 519 nm emission. The amount of Dex-FITC was quantified using a standard calibration curve in the range of 0.4 μg/mL to 18 μg/mL. The percentage of Dex-FITC released was reported as mean ± S.D. (n = 4). To establish the effect of NDs on the release of Dex-FITC, the experimental data from the two systems were regressed to fit a first-order diffusion model based on Fick’s Law equation:

3 $$\frac{{M}_{t}}{{M}_{\infty }}=1-\exp (-\frac{ADKt}{VL})$$

where Mt and M_∞_ are the amount of the drug released at time t and time infinite, A is the surface area of the gel, D is the diffusion coefficient of the drug, K is the partition coefficient, V is the volume and L is the gel thickness. In addition, the experimental data were fit to a Korsmeyer-Peppas model^58^ following this equation:

4 $$\frac{{M}_{t}}{{M}_{\infty }}=k{t}^{n}$$

where k is the release rate constant and n is the release exponent.

### 3D osteogenic differentiation studies in GelMA nanocomposite hydrogels

Four different groups were investigated to evaluate hASCs osteogenic differentiation namely: i) GelMA hydrogels without NDs or Dex, ii) GelMA-ND (0.2% w/v) nanocomposite hydrogels without Dex, iii) GelMA containing Dex as a free drug at the concentration of 1 µM and iv) GelMA nanocomposite hydrogel including the ND-Dex complex containing an amount of Dex equal to 1 µM. In all systems, hASCs were suspended in GelMA polymeric solution at the concentration of 1 × 10^6^/mL. 10 µL of each polymer solution containing hASCs were gently pipetted on a petri dish with 100–250 μm thick spacers. A glass slide treated with TMSPMA was placed on top of the pre-polymer solution, and the samples were photo-irradiated under UV light at 6.9 mW/cm^2^ for 60 seconds in sterile conditions. The glass slides containing the thin hydrogels encapsulating hASCs were transferred to a 24-well plate with fresh α-MEM, supplemented with 15% of FBS and 1% of penicillin/streptomycin. Encapsulated cells were allowed to grow in the gels for the first seven days followed by a change in the culture condition to osteoconductive media containing 10% FBS, 50 µM ascorbic acid-2 phosphate (Sigma Aldrich, USA) and 10 mM β-glycerophosphate (Sigma-Aldrich, USA) for the rest of the study in the groups containing Dex and the ND/Dex complex. At 14 days gels were stained for the presence of ALP following the standard protocol provided by the manufacturer (Alkaline Phosphatase kit, Sigma Aldrich, USA). The enzyme activity was quantified by a *p*-nitrophenyl phosphate (pNPP) colorimetric assay (Sigma-Aldrich, USA). Results were normalized to the DNA content of each gel. Briefly, samples were washed using PBS and lysed with lysis buffer. Then, they were freeze-thawed once, sonicated and centrifuged at 8,000 rpm for 10 minutes. The DNA content of the supernatant was quantified using a PicoGreen kit (Invitrogen, USA). For Alizarin Red S staining, at 14 and 21 days, cells were fixed in 4% paraformaldehyde, washed with PBS three times, then incubated with 2% Alizarin Red S (Sigma-Aldrich, USA) for 20 minutes. Brightfield images of the stained samples were taken after washing with PBS three times to remove any excess of staining. Calcium in each nanocomposite group was quantified by first determining the wet weight of each gel then freezing and lyophilizing the samples. The nanocomposites were then allowed to vortex overnight in 0.5 M hydrochloric acid for homogenization. The resulting supernatants were analyzed for calcium content via a colorimetric assay (Sigma-Aldrich, USA) per the manufacturer’s protocol, then normalized to the wet weight of the hydrogel (µg/mg).

Osteocalcin staining was carried out as further confirmation of osteogenic differentiation of hASCs in the GelMA nanocomposite system. Hydrogels were treated with 0.1% Triton X (Bio-Rad, USA) solution for 10 minutes to permeabilize the cells, followed by treatment with 10% goat serum blocking solution. The cells were then incubated with a mouse monoclonal anti-human osteocalcin (1: 100) followed by incubation with a secondary antibody Alexa Fluor 594 goat anti-mouse (1: 500) (Thermo-Fischer Scientific, USA). hASCs were counterstained with diamidino-2-phenyindole dilactate (DAPI) (Thermo-Fischer Scientific, USA) for visualizing the nuclei. The samples were imaged using a fluorescence microscope (Zeiss, Germany).

Finally, qPCR analysis was carried to evaluate the expression of several osteogenic genes including *ALP*, *SPP1*, *BGLAP* and *RUNX2*, following the same procedure described above and using *ACTB* as the control gene. mRNA from the different samples were extracted at day 7, day 14 and day 21. For each group three different samples were tested (n=3). The fold increase expression was calculated using the ΔΔCt relative method and data were normalized based on the level expression of genes found in the group of hASCs cultured on gel in basal media at day 7. Additionally, as positive control Ctrl (+), cells were grown in 24 well plate up to confluency for 7 days and then differentiated in osteoinductive media containing α-MEM, supplemented with 10% of FBS and 1% of penicillin/streptomycin, 50 µM ascorbic acid-2 phosphate, 10 mM β-glycerophosphate and 10 nM of Dex.

### Statistical analysis

Statistical analysis was performed using two-way analysis of variance (ANOVA) followed by Tukey’s multiple comparison test used to determine whether a significant difference exists between specific groups. All statistical analyses were carried out with Graphpad Prism Software 6. A p value less than 0.05 indicates statistical significance, which was displayed as *p < 0.05, **p < 0.01, ***p < 0.001.

## Electronic supplementary material

Supplementary information 

Movie S1

Movie S2

Movie S3

Movie S4
