# Supplementary material for: Controlling Adult Stem Cell Behavior Using Nanodiamond-Reinforced Hydrogel: Implication in Bone Regeneration Therapy
Source: Sci Rep. 2017 Jul 26;7:6577. doi: 10.1038/s41598-017-06028-y (PMC5529511; doi:10.1038/s41598-017-06028-y)
Supplement: Supplementary file 1 — Supplementary information [file 41598_2017_6028_MOESM1_ESM.pdf]

Supporting information

Controlling Adult Stem Cell Behavior Using Nanodiamond-Reinforced Hydrogel: Implication in Bone Regeneration Therapy

Settimio Pacelli[1], Ryan Maloney[1], Aparna R.Chakravarti[1], Jonathan Whitlow[1], Sayantani Basu[1], Saman Modaresi[1], Stevin Gehrke[2], Arghya Paul[1*]

[1] BioIntel Research Laboratory, Department of Chemical and Petroleum Engineering, School of Engineering, University of Kansas, Lawrence, KS 66045, United States.

[2] Department of Chemical and Petroleum Engineering, University of Kansas, Lawrence, KS 66045, United States

E mail corresponding author: arghya.paul@gmail.com

S. Pacelli and R. Maloney contributed equally to the work.

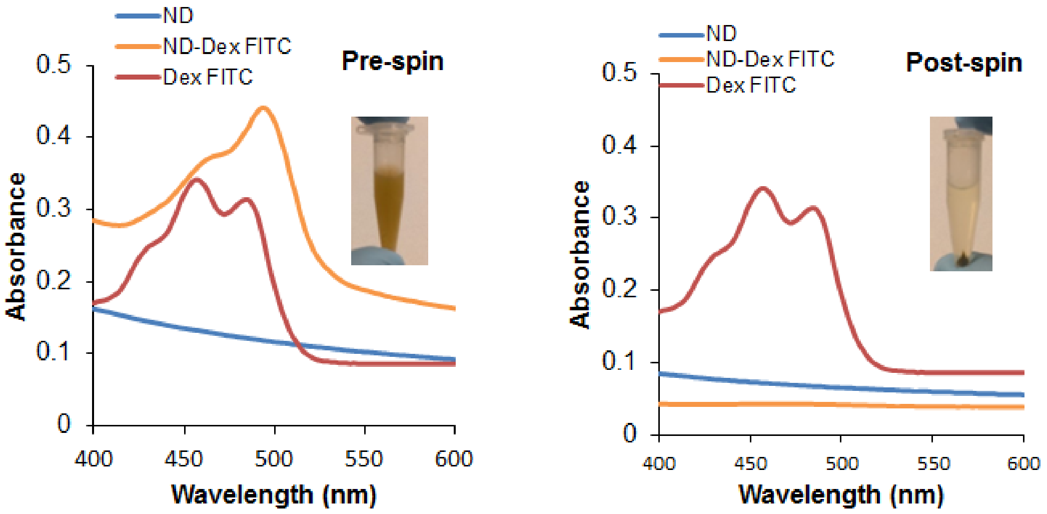

Figure S1. Visible spectra pre and post-centrifugation in the range of 400-600 nm showing the disappearance of the peak relative to the ND-Dex-FITC complex compared to the solution of DEX-FITC which displayed the same absorbance pre and post-centrifugation.

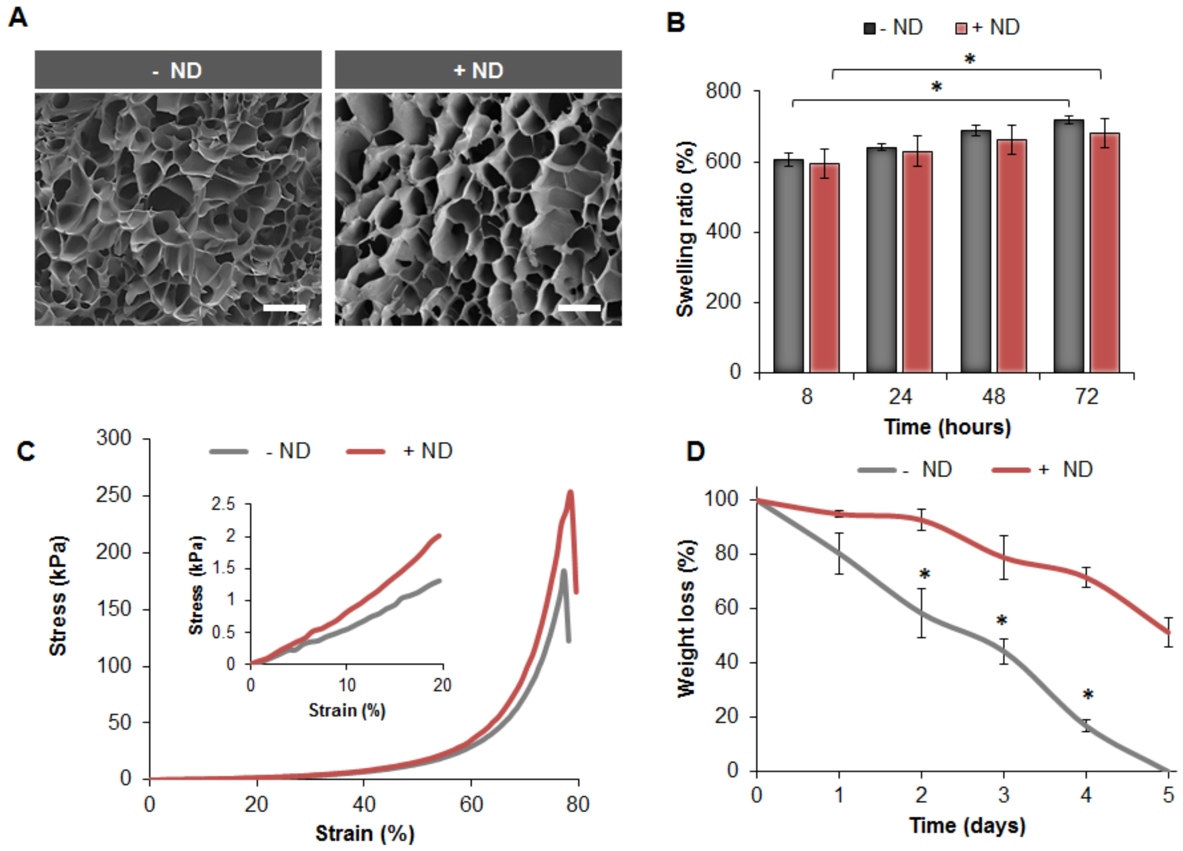

Figure S2. Influence of NDs on the physical and mechanical properties of GelMA hydrogels. **A)** SEM images of GelMA scaffold with and without NDs showing similar porosity. (Scale bar = 100 μm). **B)** The swelling ratio of nanocomposite hydrogels containing 0.2% w/v of NDs (+ND) compared to conventional GelMA hydrogels (-ND). Results are reported as mean ± S.D, (n = 5) (* = $p < 0.05$). **C)** Representative stress-strain curves obtained compressing the hydrogels with and without NDs up to the point of breakage. Insert reporting the stress-strain curves up to 20% of strain displaying an increasing in stiffness for the nanocomposite hydrogels containing 2 mg/mL of NDs (+ND). **D)** Degradability profiles obtained soaking the samples in collagenase type IV (0.5 U/mL) over time. Samples containing NDs (+ND) present lower degradability compared to GelMA hydrogels (-ND). Results are reported as mean ± S.D, (n = 5) (* = $p < 0.05$).

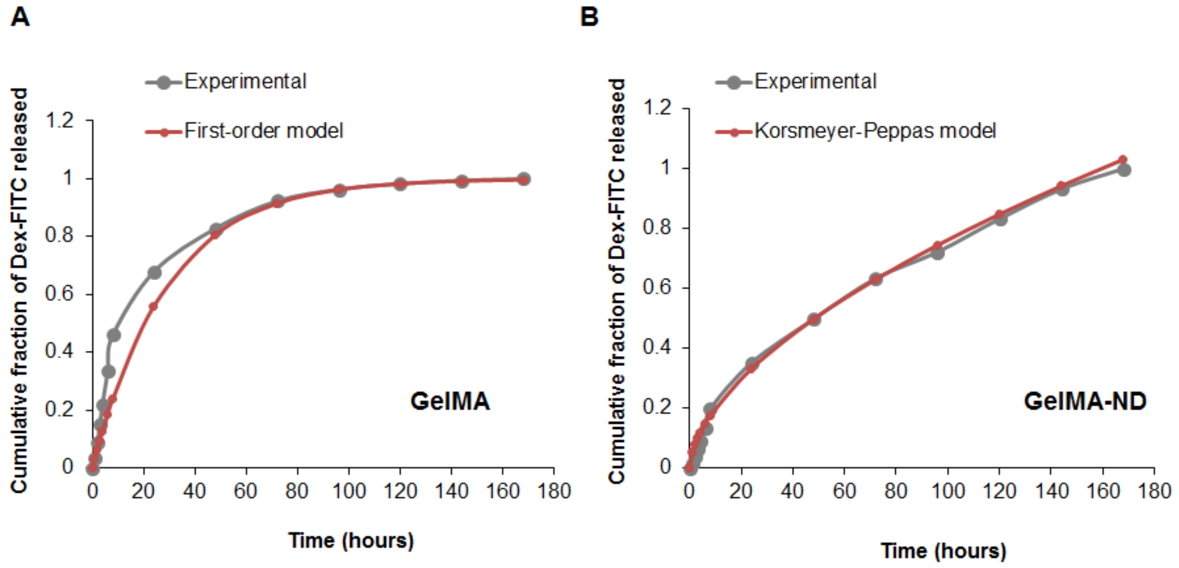

Figure S3. Comparison between experimental data and model profiles of Dex-FITC. **A)** Dex-FITC releasing from the GelMA hydrogels fits the first-order model**. B)** Dex-FITC releasing from the GelMA-ND nanocomposite systems fits the Korsmeyer-Peppas model.

Table S1. Model parameters fit to Dex-FITC experimental data

Group	Korsmeyer-Peppas model			First-order model	
	k	n	R^2	k	R^2
**GelMA**	0.1782 ± 0.0400	0.3614 ± 0.0427	0.9385	0.0342 ± 0.0014	0.9936
**GelMA-ND**	0.0525 ± 0.0036	0.5776 ± 0.0131	0.9962	0.0210 ± 0.0002	0.8883

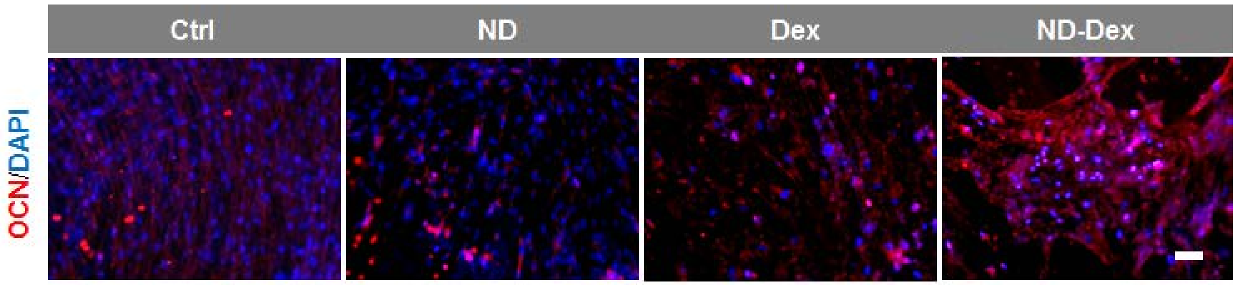

Figure S4. Osteocalcin (OCN) and Dapi staining of hASCs encapsulated in GelMA hydrogels after 21 days of culture in osteoconductive media. The reported groups are respectively GelMA hydrogels without any NDs (Ctrl), the nanocomposite GelMA/ND system 0.2% w/v (ND), the GelMA hydrogels containing only the drug (Dex) and the nanocomposite including the ND-Dex complex (ND-Dex). (Scale bar = 100 µm).
